# New insights on CRISPR/Cas9-based therapy for breast Cancer

**DOI:** 10.1186/s41021-021-00188-0

**Published:** 2021-04-29

**Authors:** Hussein Sabit, Shaimaa Abdel-Ghany, Huseyin Tombuloglu, Emre Cevik, Amany Alqosaibi, Fatma Almulhim, Afnan Al-Muhanaa

**Affiliations:** 1 Department of Genetics, Institute for Medical Research and Consultations, Imam Abdulrahman Bin Faisal University, P. O. Box: 1982, Dammam, 31441 Saudi Arabia; 2 Department of Environmental Biotechnology, College of Biotechnology, Misr University for Science and Technology, P. O. Box 77, Giza, Egypt; 3 Department of Biology, College of Science, Imam Abdulrahman Bin Faisal University, P. O. 4 Box, Dammam, 1982 Saudi Arabia; 4 Breast Imaging Division, KFHU, Imam Abdulrahman Bin Faisal University, P. O. 4 Box, Dammam, 1982 Saudi Arabia

**Keywords:** CRISPR/Cas9, Genome editing, Breast Cancer, Therapy

## Abstract

CRISPR/Cas9 has revolutionized genome-editing techniques in various biological fields including human cancer research. Cancer is a multi-step process that encompasses the accumulation of mutations that result in the hallmark of the malignant state. The goal of cancer research is to identify these mutations and correlate them with the underlying tumorigenic process. Using CRISPR/Cas9 tool, specific mutations responsible for cancer initiation and/or progression could be corrected at least in animal models as a first step towards translational applications. In the present article, we review various novel strategies that employed CRISPR/Cas9 to treat breast cancer in both in vitro and in vivo systems.

## Introduction

**Fig. 1:**
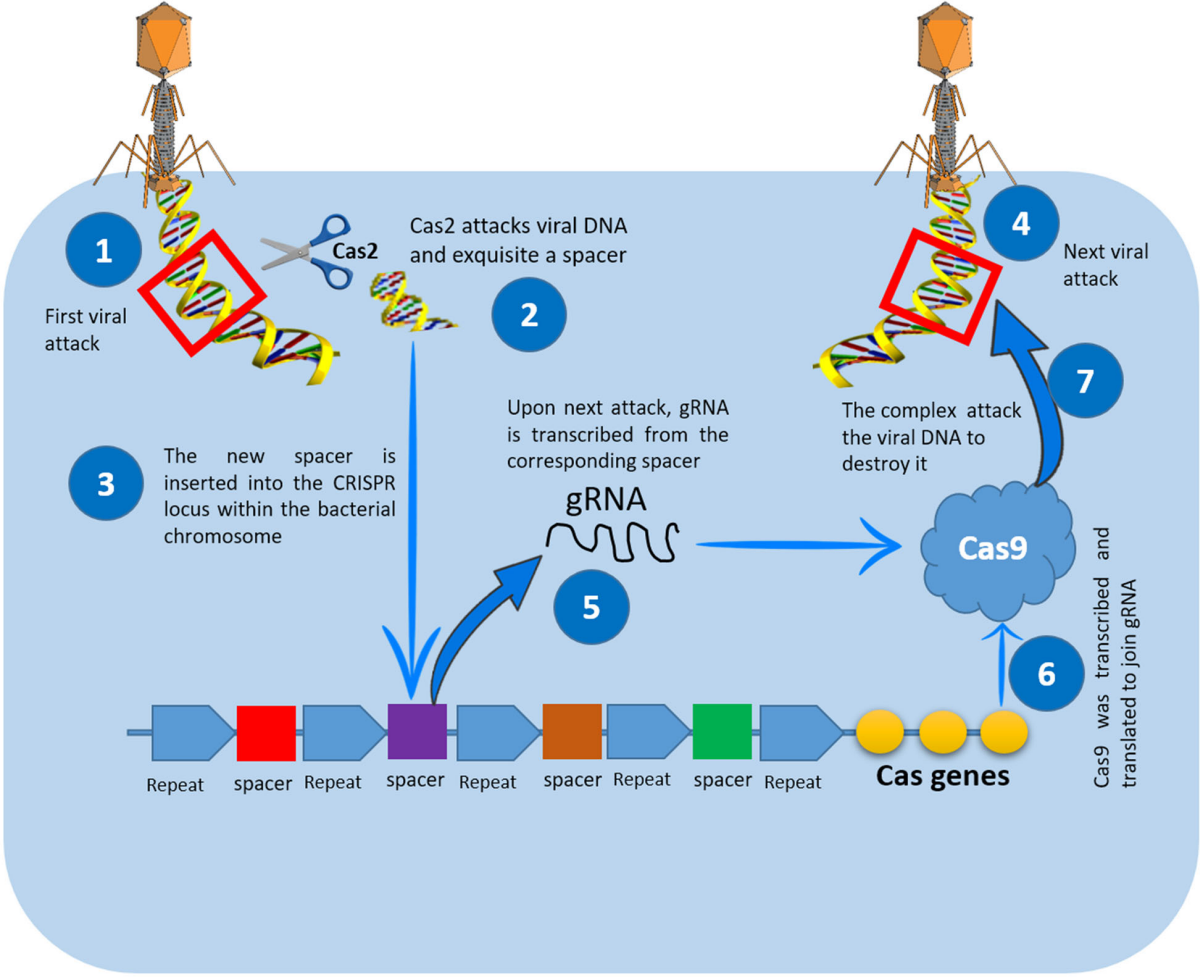
How CRISPR/Cas9 works as immune system in bacteria. When the invader (plasmid or virus) enters bacteria [1], it directs a nuclease called Cas2 to snip a short sequence of the viral genome (spacer) [2] and insert it between two repeats in its CRISPR locus [3]. When this invader type come again [4], the bacteria transcribe its spacer to generate crRNA [5], which will be matured by tracrRNA. Both types of RNA associated with Cas9 [6] will be directed to the invader genome to cleave it (using Cas9) after recognizing it (using crRNA) [7]

During the past 20 years, several genome-editing technologies have been employed in a wide range of applications. Inspired with bacterial immune system, CRISPR/Cas9 came into existence as a revolutionizing powerful tool that facilitates correction, insertion, or deletion of genetic material both in vitro and in vivo systems. The discovery of this captivating bacterial immune defense mechanism resulted in an unprecedent revolutionary change in medical sciences [1] (Fig. 1). Upon transfecting cells, Cas9/gRNA complex can find its way to the target sequence (with the help of gRNA) to delete or insert a segment of DNA (with the aid of Cas9 enzyme) [2]. This triggers the cellular endogenous repair mechanisms, which might be one of two; first: non-homology end joining (NHEJ), which is an error-prone mechanism that generates indels and can be used to disrupt a specific gene. Second: homology-directed repair (HDR) [3, 4]. When appropriately designed, a donor DNA could be inserted in the cleavage site to serve as a template on which the broken strand being built. The inserted strand might be normal or even contain a targeted mutation.

**Fig. 2:**
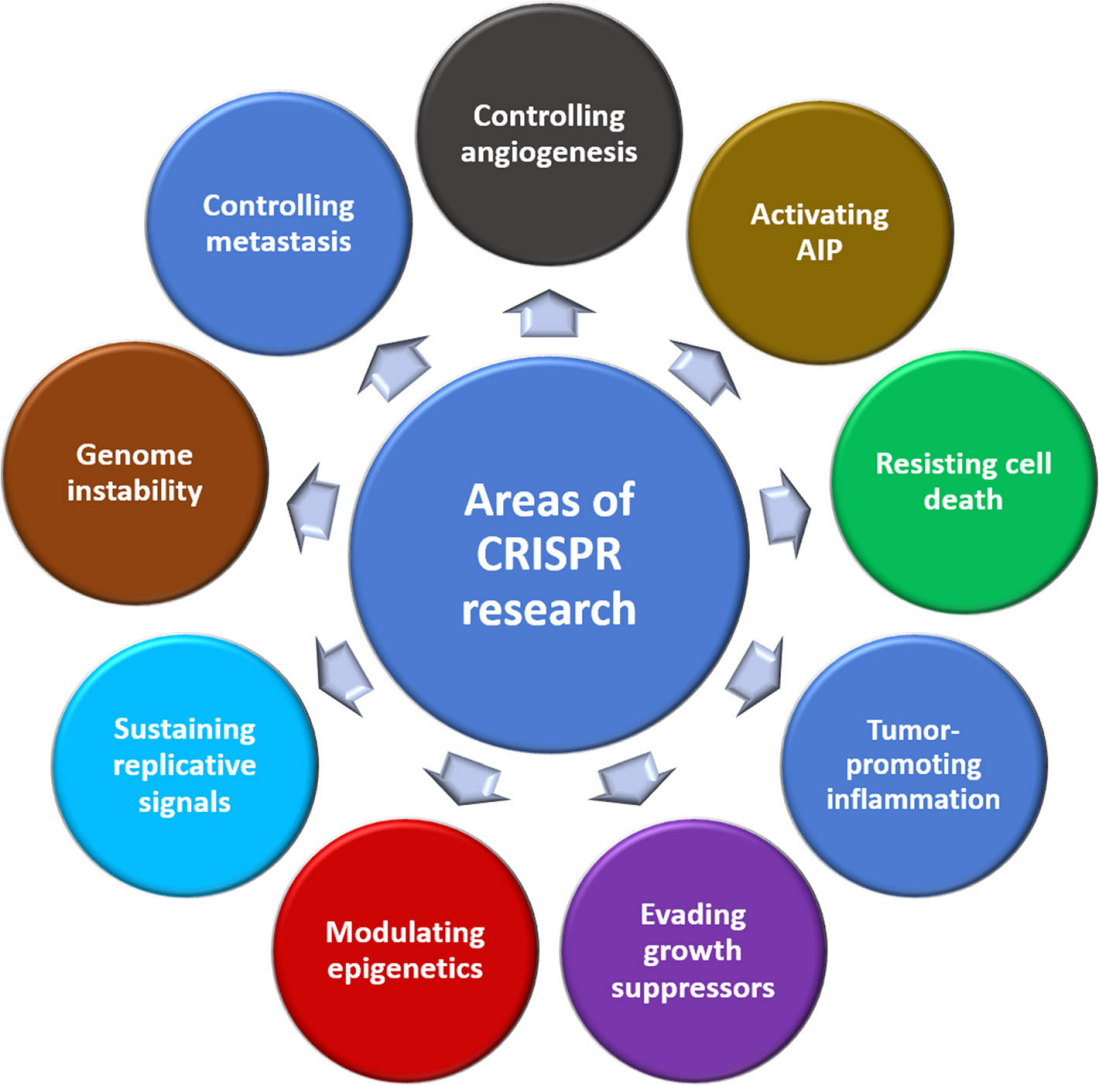
Different research and treatment areas of CRISPR/Cas9 in breast cancer

Breast cancer is a leading cause of death in females worldwide [5, 6]. Breast cancer rate is rising worldwide with an expansion in forceful neoplasia in women. Around half of the breast malignancy cases and 60% of the deaths are happening in developing countries. There is an extensive distinction in breast cancer rate among Hispanic, Caucasian and Asian women, with Caucasian women being the most astounding and Asian women being the least [7, 8]. Breast cancer research is a hot area in which CRISPR technology is in the core (Fig. 2).

CRISPR/Cas9 takes its place as an essential, efficient, and straightforward tool for cancer research, especially breast cancer [9]. This tool provides a means to insert, correct, and remove the faulty genes in a precise manner. Some limitations are facing applying this technology in all types of cancer; however, advancements are taking place [10].

## CRISPR/Cas9: the Nature’s gift

CRISPR is primarily a gift from the Mother Nature, where scientists discovered it as prokaryotic immune system in bacteria and archaea. The work on CRISPR starts in the late 1980s with several landmarks in this long journey (Table 1), however, the foundational publications came in 2012 to demonstrate that a CRISPR system in *Streptococcus pyogenes* could be used for genome editing, opening a new gate for genome engineering. In these bacteria, Cas9 is responsible for cleaving the invaders’ DNA. Guided by crRNA (CRISPR RNA) and tracrRNA (trans-activating crRNA which will be combined for editing purposes in vitro to produces gRNA), Cas9 can target a specific site in the genome and then produces double strand breaks (DSBs) [32, 33]. The complex composed of Cas9 and gRNA attacks the specific DNA sequence just upstream of the protospacer-adjacent motif (PAM) sequence, NGG (N represents any nucleotide) [34, 35]. Almost 60 and 40% of archaea and bacteria, respectively used CRISPR in the same manner, with minor differences [36]. CRISPR location, in this sense, serves as a memory deposition where the bacteria can store previous viral or plasmid attacks, and then used this information to defend itself against these invaders in the upcoming attacks.

**Table 1:** The timeline of genome editing groundbreaking achievements

Year	Contributor	Contribution
**1987**	Yoshizumi Ishino et al.	Discovered some repeats in the *IAP* gene in *Escherichia coli*. They could not identify the main function of these repeats [11].
**1993**	Francisco Mojica et al.	Characterized what is now called a CRISPR locus as a molecular genetics’ memory of MGE (mobile genetic element) that previously attacked the bacteria cell. They also discovered tandem repeats (TREPs) in Haloarchaea [12].
**1996**	Yang-Gyun Kim et al.	Genetically engineered the first restriction-modification enzymes with the ability to cut at specific target sequences. This enzyme was amenable to be used in editing genomes [13].
**2000**	Jeff Smith et al.	Conducted several experiments to show that Zinc Finger Nucleases (ZFN) can generate double-stranded DNA breaks, and hence could be used as a genome-editing tool [14].
**2002**	Marina Bibikova et al.	Used Zinc fingers for the first time to disrupt genes in the fruit fly *Drosophila melanogaster* [15].
**2002**	Ruud Jansen et al.	Coined the term CRISPR that stands for Clustered Regularly Interspaced Short Palindromic Repeats [16].
**2004**	Alan Lloyd et al.	Announced the first plant genome modified with the Zinc Finger Nuclease, that is the tool that could be used in editing genomes. This tool could also be used to create models of human genetic diseases [17].
**2005**	Alexander Bolotin et al.	Discovered a new CRISPR locus in *streptococcus thermophilus*. He also noted that the spacers, which have homology to viral genes, share a common sequence at one end. This sequence would be later known as protospacer adjacent motif (PAM) [18].
**2006**	Eugene Koonin et al.	Suggested that CRISPR is an immune system that works by interference with RNA in bacteria. He also classified about 25 distinct Cas families, and predicted their new functions [19].
**2008**	John van der Oost et al.	Showed that spacer sequences are transcribed into CRISPR RNAs (crRNAs), that guide Cas nuclease to the target DNA of the invader [20].
**2010**	Sylvain Moineau et al.	Indicated that CRISPR/Cas9 generates DSBs 3 nucleotides upstream of the PAM sequence in target DNA [21].
**2011**	Emmanuelle Charpentier et al.	Discovered another type of RNA found in the CRISPR system’s components called trans-activating RNA (tracrRNA). She indicated that tracrRNA works simultaneously with crRNA in directing the Cas9 protein to cut the target sequence [22].
**2012**	Virginijus Siksnys et al.	Indicated that Cas9 consists of two domains, which are RuvC and HNH, and that the first domain cuts in the distant DNA strand, while the second cuts in the strand where the integration between crRNA and DNA occurs [23].
**2012**	Jennifer Doudna and Emmanuelle Charpentier	Engineered the dual-tracrRNA:crRNA as a single RNA chimera called gRNA [24].
**2013**	Feng Zhang et al.	Announced the use of CRISPR/Cas9 in the editing of human genes, the matter that opened the door to the use of CRISPR in the medical field [25].
**2015**	Feng Zhang et al.	Introduced Cpf1 as a new nuclease that works in more efficient way than Cas9 [26].
**2015**	Junjiu Huang et al.	Reported the first application of CRISPR to non-viable human embryos [27].
**2016**	Kamel Khalili et al.	Used CRISPR/Cas9 to edit HIV out of a human immune cell DNA, and therefore, prevent the reinfection of unedited cells too [28].
**2016**	Kevin Esvelt et al.	Developed the CRISPR/Cas9 gene drive [29].
**2017**	Jennifer Doudna et al.	Developed a CRISPR-Gold, which is a new version of the CRISPR/Cas9 gene editing, in this new technology, they used gold nanoparticles for delivering the CRISPR/Cas9 gene-editing system into cells [30].
**2018**	Norbert Reich et al.	Introduced light-triggered genome editing approach using hollow gold nanospheres. This approach is 100 to 1000 times more efficient than current genome editing methods [31].

### CRISPR/Cas9-based tumor modeling

The classical method to transform a normal cell into malignant one requires multistep process that involve series of mutations to acquire such cells the hallmarks that characterize the malignant phenotype [37–39]. With exploiting the power of CRISPR/Cas9, many research groups were able to create such cancer models (Table 2). Truncating *APC* tumor suppressor gene in gut cells was successful representative for a well-developed early event in colorectal cancer development. The *Wnt* signaling activators have been removed from the culture medium to select *APC*-lacking human intestinal stem cells, leading to provoked *β*-catenin stabilization, and up-regulation of *Wnt* pathway [48] and [49]. Other approaches involve creating cells with activated *KRAS* oncogene along with loss-of-function mutation. Furthermore, *P53* was also inactivated using CRISPR/Cas9 [50, 51]. Using these approaches, a combination of mutations could also be generated, and then tested in by inoculating these edited cells into immunodeficient mice. This cancer model answers an immense question, whether the cancer-causing mutation occur randomly or in a precise time order.

**Table 2:** CRISPR/Cas9- based cancer mouse models (reviewed in [40])

Cancer type	Mouse strain	Vector	Target gene(s)	Reference
Lung adenocarcinoma	*Kras*^*lsl*-G12D/+^	Lentivirus	*NKX, PTEN, APC*	[41]
Lung adenocarcinoma	CD1	Adenovirus	*EML4*	[42]
Burkitt lymphoma	Arf/− EμMyc	Retrovirus	*p53*	[43]
Glioblastoma	Crl:CD1 mice	Transfection	*TRP53, PTEN, NF1*	[10]
Acute myeloid leukemia	C57Bl/6 mice	Lentivirus	*DNMT3A, EZH2, RUNX1*	[1]
Burkitt lymphoma	Eμ--Myc mice	Lentivirus	*p53*	[44]
Pancreatic adenocarcinoma	*Kras*LSL-G12D/+	Lentivirus	*LKB1*	[45]
Lung metastases	*Kras*^G12D^/+; *p53*^−/−^	Lentiviral	Multiple hits	[46]
Tumor regression	*Foxn1*^nu^mice	Transfection	PKCβ A509T	[47]

Similar strategies have been used to transduce a non-metastatic mouse lung cancer cell line with CRISPR/Cas9 which targets several protein-coding genes along with miRNA precursors. Chen, Peng [52] recorded a massive growth of tumor and lung metastasis upon inoculating the modified cells into immunodeficient mice. By deep sequencing, the team discovered several novel genes whose activation was crucial to tumor growth and metastasis. This approach allows for recapitulating the process of tumor evolution and metastasis, which in turn, might help in designing specific therapies targeting the faulty gene(s) [53].

### CRISPR/Cas9-based transcriptome reprogramming

Transcriptional programs control almost all aspects of organism’s life from early developmental stages until death [54]. Cancer, as an abnormal state of the cells has its own specific transcription program, which has been validated by CRISPR in several cancer types including ovarian, and breast carcinomas, where cancer cells were found to display a cell-specific transcription regulation pattern, with epigenetic modulations being the main affecting factors [55, 56]. This program could be disrupted by inhibiting *CDK7*, which represents a potential therapeutic option for breast cancer, especially estrogen receptor (ER) mutation-mediated endocrine-resistant type. The most impressive feature is that *CDK7* inhibition not only prejudices the cell growth of breast cancer, but also the resistance of the estrogen-responsive MCF7 breast cancer cells [57].

Prior CRISPR, ZFNs (zinc finger nucleases) and TALENs (transcription activator-like effector nucleases) were used to activate and suppress genes as a strategy for cancer therapy. A brief comparison between the three major genome editing tools is highlighted in (Table 3). The structure of both tools made them suitable for holding activators and suppressors [58, 59]. For the time being, these activators and suppressors are easily fused to dCas9, a mutation of Cas9 without endonuclease activity. Recently, more dCas9-based transcription activator/suppressors were made available [60]. Aptamers been used for re-programming the epigenome aiming to reactivate the hypermethylated tumor suppressor genes (TSGs) to recover the growth suppressor activity exerted by these TSGs [61, 62]. This strategy works in liver, colon, breast, and lung cancers. This approach is an alternative to epigenetic-modified small molecules/drugs, which might have undesirable side effects. Furthermore, dCas9 was efficiently used for targeted demethylation of *BRCA1* promoter using the demethylation domain of *TET1*—the enzyme that converts 5-mC (5-methylcytosine) into 5-hmC (5-hydroxymethylcytosine)— in cervical and breast cancer cells. CRISPR/Cas9-based epigenome editing was used also to repress interleukin receptors (*IL1R1*) and tumor necrosis factor *α* receptor (*TNFR1*) in human adipose-derived stem cells. This may open the gate to control various kinds of inflammations that accelerate the growth of different types of cancers [63–65].

**Table 3:** Comparison between the three main genome-editing tools

	ZFN	TALEN	CRISPR
Nature	Engineered protein to target specific DNA sequences	Engineered protein to target specific DNA sequences	Short sequence of RNA that can target specific DNA sequences
Target/sensitivity	Protein–DNA interaction, less sensitive	Protein–DNA interaction, less sensitive	RNA–DNA interactions, highly sensitive
Size of recognized target	18–36 nt	30–40 nt	22 nt
Off targeting	High	Moderate	Low
Efficiency	Moderate	Moderate	High
Nuclease – D/M	FokI – dimer	FokI – dimer	Cas9 – monomer
Mode of action	For ZFN to work, it requires two sets to hybridize each to each DNA strand around the target sequence	For TALEN to work, it requires two sets to hybridize each to each DNA strand around the target sequence	In the presence of gRNA, Cas9 can reach the target DNA sequence and generate double strand breaks.
Cytotoxicity	Moderate	Low	Low
Multiple targets	Difficult	Difficult	Doable
Cost/benefits	High cost and time consuming	High cost and time consuming	Low cost and less time needed

Yet, an array of epigenetics modifiers based on dCas9 is introduced to modify the epigenetics marks as a new way to treat several diseases including cancer.

### Editing DNA for cancer therapy

Although CRISPR technology was used in cancer modeling and screening, it also offers a straightforward way to target-specific cancer therapy. Many trials have been conducted using CRISPR to combat this life-threatening disease. *E6* and *E7* in the HPV have been challenged with CRISPR/Cas9 to induce the apoptotic machinery and inhibit growth in cervix cancer cell line in vitro [66]. Meanwhile, CRISPR/Cas9-mediated deletion of miRNA-binding site located in the UTR (un-translated region) of *F1H1*—the gene that regulate angiogenesis in NSCLC cells— resulted in recovering the vascular abnormalities that characterize lung cancer [67].

The oncogene *HER2* exons are also a target for CRISPR intervention, where a mutation in the exon12 of *HER2* resulted in a dominant negative mutant phenotype [68]. The mutant *HER2* was found to inhibit the *MAPK*/*ERK* axis of *HER2* signaling pathway required for the proliferation of breast cancer cells. Controlling breast cancer via CRISPR-mediated editing of *HER2* is enhanced in the presence of *PARP* inhibitors that is involved in DNA repair and cell death [69]. This combinational approach represents a potential therapeutic option for breast cancer.

Genome-wide synthetic lethal CRISPR screens were performed to determine novel as a therapeutic option for treating endocrine resistant breast cancer. CRISPR screens identified a gene which is associated with the response to endocrine therapy in plentiful of clinical studies [70].

To this end, they tried to combine two therapeutic components; ordinary endocrine therapy and an inhibitor for the gene identified by the CRISPR screens to subdue the endocrine resistance either in cell line or patient-derived xenograft models.

### Protein degradation in breast cancer

One of the important ways to enhance tumor cell proliferation is the increased activity of protein degradation. Of these protein-degrading enzymes, comes the 26S proteasome, a multi-catalytic enzyme that is responsible for protein degradation including cell cycle regulation and apoptosis-related proteins [71, 72]. In cancer models, it has been indicated that proteasome inhibitors have anticancer and apoptosis-enhancing properties. Furthermore, it sensitizes tumor cells to the extrinsic and intrinsic pro-apoptotic signals. Therefore, proteasome has become a target for antitumor treatments. It has been indicated that breast cancer proliferation is controlled by a site-specific proteasome phosphorylation process [24], and the interfering and disruption of this process might be of value in controlling the disease.

Using CRISPR/Cas9, dual-specificity tyrosine-regulated kinase 2 (DYRK2) knockout (the enzymes that phosphorylate the proteasome components) was established to disrupt tumorigenesis of the proteasome-addicted human breast carcinoma cells in mice [73].

ER-positive breast cancer could be treated via inhibition of estrogen synthesis using aromatase inhibitors (AI) or via tamoxifen and fulvestrant that compete estrogen on ER*α*. Mutations in ER*α* such as ER*α*Y537S and ER*α*D538G results in rendering advanced metastatic breast cancer insensitive to AI and tamoxifen [74]. To validate the effect of these mutations, breast cancer positive ER*α* model was created using CRISPR/Cas9 in which the wild type version of ER*α* was replaced with ER*α*Y537S or ER*α*D538G. These mutant cells are partially resistant to antiestrogen, i.e., it is manifesting estrogen independent. Antiestrogen resistance of the mutant cells was found to be associated with upregulation of the protein response that reduces the ER*α* degradation [75].

Migration and invasion enhancer (*MIEN1*) are involved in cancer progression and metastasis of breast cancer. It has been found that increased expression of *MIEN1* might enhances tumor migration and metastasis. Using CRISPR/Cas9, a targeted deletion in this gene effectively led to repeal its expression and hence to control the disease spearing. This approach allows us to deeply understand the role *MIEN1* plays in carcinogenesis and tumor progression, which might be transformed in the future as a breast cancer therapeutic option [76].

Mutations in *PTEN* (phosphatase and tensin homolog) gene*,* are fundamental step in the carcinogenesis process. *PTEN* is a tumor suppressor gene, involved in the cell cycle regulation and controlling the proliferation rate. Using CRISPR/Cas9, invasive lobular breast carcinoma (ILC)-initiating cells was targeted to disrupt *PTEN* in mice mammary gland-specific loss of E-cadherin. This approach can be used for rapid in vivo testing of putative tumor suppressor genes underlie ILC [77].

### CRISPR-mediated immunotherapy

**Fig. 3:**
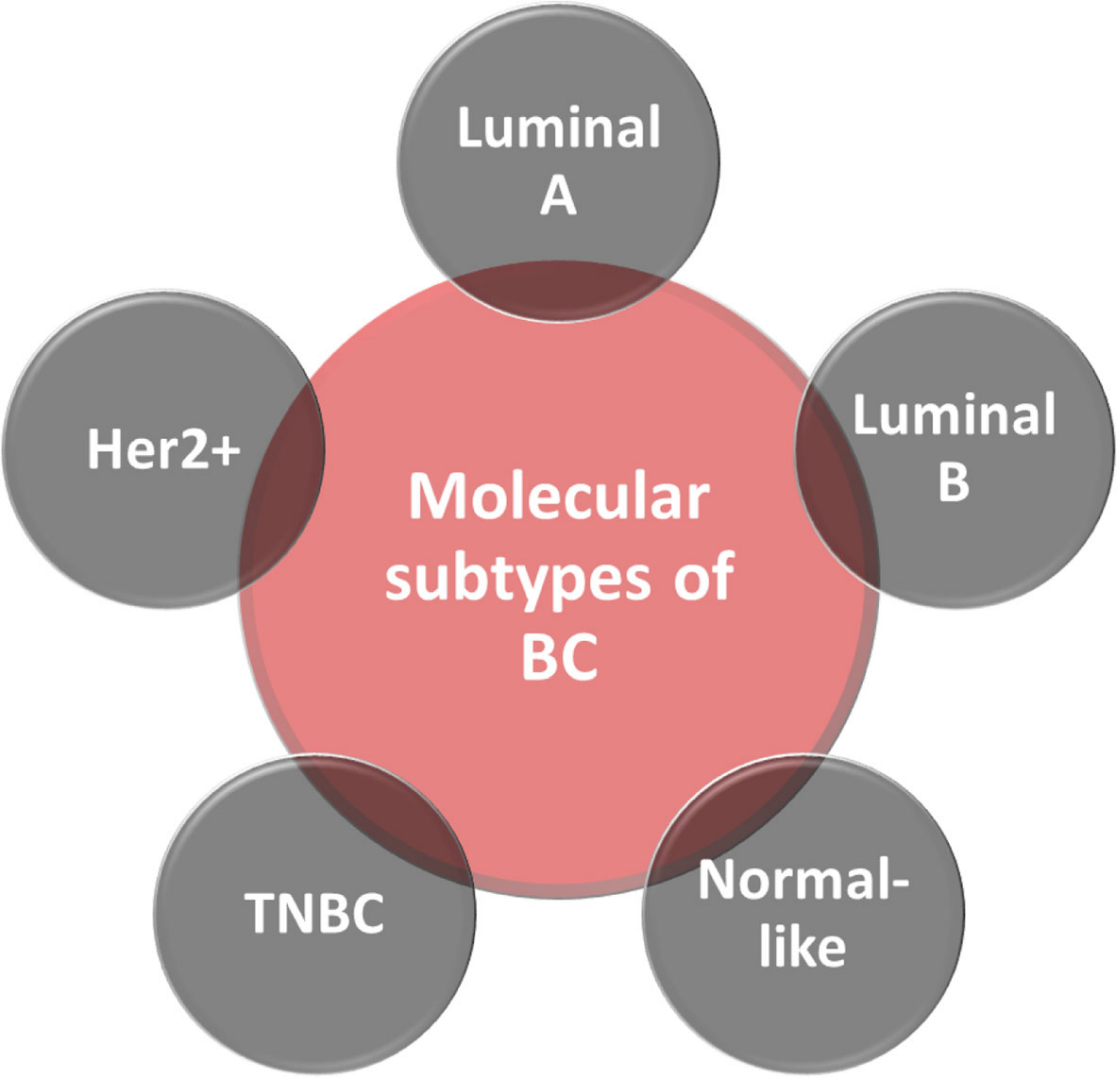
The molecular subtypes of breast cancer

Molecular subtypes of breast cancer have different characteristics based on which cancerous cells respond to the intrinsic (immune system) or extrinsic (environment) stimuli (Fig. 3). One unique approach to fight breast cancer cells is using a CRISPR/Cas9-edited macrophages in which signal-regulatory protein alpha (SIRP-*α*) is eliminated. The edited macrophages will be unable to receive the “do not eat me” signal of CD47-SIRP*α* from the cancer cells leading to destroy it. On the other hand, T cells could be engineered to express a cancer-specific T-cell receptor (TCR). Taking into consideration that the endogenous TCRs might compete with engineered one, endogenous TCR-*β* was knocked out in the recipient cells via CRISPR/Cas9. The resultant CRISPR-edited T cells were a 1000-fold more sensitive to cancer antigen than normal TCR-transduced T cells [78]. Meanwhile, CAR T cells were edited by CRISPR/Cas9 to generate inhibition-resistant universal CAR T cells [79].

### Editing cancer epigenome

CRISPR/Cas9-mediated epigenome editing is one of the potential tools to treat several cancers including breast cancer. The gene approach involves the fusion of Cas9 with a transcription repressor/activator for repression and activation, respectively. The Krüppel-associated box (KRAB) is an example, where the fused dCas9-KRAB was capable to induce locus-specific deposition of H3K9me3 at the HS2 enhancer region, resulting in silencing of multiple globin genes in K562 cells [80]. Cancer cells normally contain a wide variety of genetic mutations either in TSGs or in oncogenes [37, 81]. Using CRISPR/Cas9, specific loss-of-function or gain-of-function mutations could be achieved. This approach enables scientists to identity the causative gene in different types of cancers including breast cancer. Targeting these genes might help in controlling the corresponding cancer. Meanwhile, The ER regulator *SRC-1* gene plays a central role in the ability of ER tumors to adapt and facilitate metastatic disease progression. *SRC-1* coactivates ER to regulate a network of proliferation- and differentiation-associated genes critical to breast cancer progression. Using CRISPR/Cas9-based epigenetic, silencing of *SRC-1* resulted in a poor expression of the proliferation- and differentiation-associated genes, which might control the progression of breast cancer and/or tumor metastasis [82].

### CRISPR barcoding

Molecular barcoding using mutations induced by Cas9 is an influential method for recording biological information on real time, however, its applications in mammalian systems is relatively limited [83]. Breast cancer progression and proper response to treatments are major aspects being affected with the large count of mutations accumulated in malignant cells [84, 85]. Although its significant clinical impact, few studies have addressed the intratumor heterogeneity to deeply understand the causative genes and along with underlying molecular mechanisms. Several research groups used CRISPR/Cas9 to introduce oncogenic mutation in a subset of cells within a mass population [86]. These targeted mutations were linked with silent mutations that serve as a genetic barcode and can be identified by qPCR. This approach has been used in different malignant cells to introduce nonsense or missense mutations in *TP53*, resulting in an inactive or a dominant-negative form of this TSG. In this context, current genome editing approaches lack the capability to generate specific alterations with tumor growth-controlling properties making it hard to repair oncogenic mutations. Initial tracing of the edited cells using CRISPR-barcoding overcomes this limitation [87–89].

## CRISPR/Cas9 limitations and challenges

### Drug resistance

One of the most important challenges in breast cancer therapy is the drug resistance [90]. Accordingly, identifying the drug resistance-related genes might set a new stage of treating breast cancer potentially by using CRISPR/Cas9 technique. The main limitation faces CRISPR application in editing these genes is the off-target activity of Cas9. Various approaches were used to tackle this problem, although the defined mechanisms of high specificity of gRNA remains unclear [91, 92].

Different methods have been introduced to screen for the edited alleles created by CRISPR/Cas9. Among these methods the “pop-in/pop-out” established by Kühn and Chu, in which the isolation of edited alleles became easier [93].

One of the biggest limitations of applying CRISPR/Cas9 for clinical treatments is the presence of a type of antigen-specific T-cells works against Cas9. This, of course, limits the activity of the nuclease and, subsequently, the effectiveness of the entire editing process. Furthermore, the immune system can recognize and eliminate the edited cells. Several reviews highlighted the potential immunological concerns of using CRISPR/Cas9 in clinical settings [3, 94–96]. However, more studies are needed to elucidate the function of Cas9-specific T-cells during treatment. These studies should also address engineering a type of Cas9 that can escape the host immune system or at least fusing an immune-compromising agent within the Cas9-harboring cassette.

Another challenge is the delivery system that suit the hard-to-transfect cells/tissues. Several non-viral delivery methods are there including using nanoparticles, electroporation, and direct injection. These methods have its own drawbacks where it needs in general large quantities of the prepared plasmids along with tools required.

Viral and bacteriophage-derived vectors represent the easiest way to deliver CRISPR/Cas9 inside the target cells, however, more preclinical studies are mandatory to characterize its level of geno- and cellular toxicity [97]. In addition, the pharmacokinetics and pharmacodynamic properties of the complex must be identified. Some trials have been conducted to effectively deliver CRISPR system into cells including the encapsulation of the system into lipopolymer with cell specific aptamer for cancer-specific targeting. This method appears to be more efficient in CRISPR system delivery compared to the traditional viral and non-viral delivery methods [98]. Furthermore, Wang, Song [4] used CRISPR/Cas9 delivery system comprising PEGylated nanoparticles based on the *α*-helical polypeptide PPABLG. Supported by the high membrane-penetrating capability of the polypeptide, P-HNPs attained competent cellular internalization and endosomal escape, which is considered an efficient way to system delivery.

### Off-targeting

The major challenge facing CRISPR/Cas9-based therapeutics is the off-target possibility, where Cas9 can cleave DNA at unintended sites [99]. To minimize the off-target effects, the design of gRNA should be of high precision, especially the 5′ end sequence composition. Therefore, several algorithms have been introduced in the recent decade to maximize the on-target and minimize the off-target (Table 4). On the other hand, the activity of Cas9 is considered a crucial defining factor that identify the off-target effect [100]. The higher the activity of Cas9, the increased the level of off-targets (due to non-specific cleavage). Thus, adjusting the Cas9 activity might help in reducing the off-target effects. To this end, various studies were conducted to optimize Cas9 activity. One of the ways to produce conditionally expressed Cas9 is to clone the Cas9-encoding gene under the control of tetracycline-responsive element promoter. This might help in controlling the expression of Cas9 in the presence of tetracycline/doxycycline. Furthermore, cloning gRNA under the same promoter was found to be a good alternative to control off-target cleavages [101–103].

**Table 4:** A group of web-based algorithm for gRNA designing

Tool name	Off-target analysis	Webpage link
CRISPR Design Tool	Yes	https://www.synthego.com/products/bioinformatics/crispr-design-tool/
Cas-Designer	No	http://www.rgenome.net/cas-designer/
CRISPR MultiTargeter	No	http://www.multicrispr.net/
CRISPR Genome Analysis Tool		http://cbc.gdcb.iastate.edu/cgat/
GeneArt	Yes	https://www.thermofisher.com/sa/en/home/life-science/genome-editing/geneart-crispr/geneart-crispr-search-and-design-tool.html/
ZiFiT Targeter Version 4.2	No	http://zifit.partners.org/ZiFiT/ChoiceMenu.aspx/
CRISPR/Cas9 target online predictor	Yes	https://crispr.cos.uni-heidelberg.de/
Cas9 online designer	Yes	http://www.rgenome.net/cas-designer/
CHOPCHOP	Yes	http://chopchop.cbu.uib.no/
sgRNAcas9	Yes	http://www.biootools.com/col.jsp?id=103/
E-CRISP	Yes	http://www.e-crisp.org/E-CRISP/

Another effective approach to control the level of Cas9 expression to decrease off-targets is constructing a version of Cas9, which is 4-hydroxytamoxifen-dependent via attaching a hormone-binding domain of the estrogen receptor to Cas9. In this case, Cas9 is released to the cytoplasm in the presence of 4-hydroxytamoxifen leading to control Cas9 activity [104]. Other approaches implicated the self-splicing properties of protein segments “intein” that can excise itself and join the remaining portions with a peptide bond to render Cas9 nuclease active only in the presence of 4-hydroxytamoxifen [34]. Interestingly, these two systems of controlling the expression of Cas9 might be used soon as a tool to design vectors for therapeutic interventions.

Furthermore, target site recognition by Cas9 requires the recognition of a specific short motif (PAM, protospacer recognition motifs). To tackle this problem, an engineered Cas9 derivatives with altered PAM specificities was designed [105]. Meanwhile, a split variant of Cas9 was fused with low expression protein domain. In this composition, Cas9 became active when stimulated with blue light, allowing researchers to control the activity of Cas9 in vitro and in vivo [106].

On-targeting is determined generally by several factors including gRNA physical design, the nuclease structure (Cas9), the ratio of gRNA/Cas9 in the media, and finally the target site uniqueness [107]. Various approaches were introduced to tackle off-targeting problem including down-sizing the gRNAs to 20 bases, which could increase its specificity by 5000-fold [105]. Other approach involves mutating the Cas9 active domain to alter its cleavage function aiming to enforce it to use two-enzyme unite to make double stand break (DSB) [108]. Furthermore, fusing dCas9 with FokI to increase the specificity was suggested [109]. Altering the electric polarity of the Cas9 two domain; HNH and RuvC to reduce off-target editing, was proved to be more specific with reduced off-target score [103]. One research group has tried to introduce the Cas9 in its protein form rather than being inserted as a Cas9-coding DNA in the plasmid might enhance the on-target capacity and reduce off-target mutations [110].

For CRISPR/Cas9 technology to be applied in clinical settings, the challenge of controlling Cas9 activity should be tackled, taking into consideration that the easy and straightforward the approach, the higher the chance to be used as a therapeutic option for many types of cancer, including breast cancer.

### Animal studies

Kleinstiver et al. [77] described a novel approach to validate candidate TSGs that have a role on invasive lobular breast carcinoma by intraductal injection of lentiviral vector that encodes CRISPR/Cas9 system, Cre recombinase, or a combination of the two components in female mice with conditional alleles E-cadherin gene. Thier work enables to identify the putative TSG implicated in invasive lobular breast carcinoma in mice.

In the same context, breast cancer-related miRNAs such as miR-23b and miR-27b were knocked out using CRISPR/Cas9 to examine the breast cancer model in vitro and in vivo. In vitro knock out of miR-23b and miR-27b revealed that these miRNAs are oncogenic miRNAs in MCF7 breast cancer cells in mice. Results also indicated that miR-27b could have tumor suppressive activity under certain circumstances [111].

A knock-in mice with Cre-conditional expression of a cytidine base editor was generated to test the utility for precise somatic engineering of missense mutations in breast cancer. A designed sgRNA-encoding vector was delivered to induce point mutation to assess the effect of defined allelic variants on mammary tumorigenesis. This model was successfully applied in a model of TNBC [112]. Other base editing trial in breast cancer was conducted by [113].

## Ethical issues

CRISPR/Cas gene editing technologies have emerged as powerful tools in the study of oncogenic transformation [114]. Although its benefits, it can raise persistent ethical concerns [115]. Recently, CRISPR/Cas9 has been picked as favored technique for genome editing because of its high level of straightforwardness along with the requirements of minimal efforts. These properties make this strategy alluring to be utilized by any molecular science lab, yet the issue is that it can be utilized for any reason except if it is managed [116].

One of the main drawbacks of this technology is the Off-target effects that might have several pathogenic consequences [115]. On the other hand, on-targets may also result in a wide range of deletions and genomic rearrangements [117].

## Future perspectives

CRISPR/Cas9 is not only a powerful tool to edit genomes in laboratories, but also it can serve as a therapeutic option after ensuring its safety profiles. Prior applying CRISPR/Cas9 directly to human cells, animal cancer models serve as a preclinical platform in which this tool could be used to create models to deeply elucidate the causative genes of such disease. Furthermore, these models could be investigated in parallel to the cancer patients, which make it easy to rapidly identify different resistance mechanisms and to establish new strategies for treating the disease. Furthermore, since Doudna and Charpentier announced that CRISPR technology could be used in lab as an RNA-guided genome editing tool, CRISPR technology became the ideal platform to personalized medicine, where it offers unique opportunity to edit human genome easily and straightforwardly in specific manner. For the time being, CRISPR capable to correct single mutation in human, and with pushing the technology to its upper limits, multiple genes could be corrected, removed, replaced, or inserted in vivo at the same time in one single hit. Applications of CRISPR cover almost all the biological and biomedicine research [41].

## Conclusion

CRISPR/Cas9 is a groundbreaking technology that can be used to cure several human diseases including cancer. In this review, we highlight the most advanced CRISPR/Cas9-based approaches to tackle the challenges associated with many types of cancer including breast cancer. Breast cancer is not only caused by genetic mutations but also by epigenetic one, making CRISPR an ideal tool to deal with mutations underlie this disease. Although CRISPR has a profound range of application especially in human diseases, some ethical issues have arisen fearing from the misuse of this technology. The consequentialist arguments mainly seek the balance between potential benefits and risks in ethical considerations. Nonetheless, the use of CRISPR/Cas9 in somatic cells is ethically accepted because of its low risk compared to its benefits. In addition, germline applications for human embryos have high risks compared to its potential benefits, where it may have unknown harmful effects on future offspring. Nevertheless, one can argue that, for example, correcting the faulty version of the *MYBPC3* gene that causes hypertrophic cardiomyopathy in fetus might help in the selection of the healthy embryos for implantation as a potential therapeutic intervention to treat monogenic inherited disorders.

## Data Availability

All data analyzed and reviewed are included in this article.

## Abbreviations

CRISPR: Clustered Regularly Interspaced Short Palindromic Repeats
Cas9: CRISPR associated protein 9
NHEJ: Non-homology end joining
HDR: Homology-directed repair
tracrRNA: Trans-activating crRNA
crRNA: CRISPR RNA
gRNA: Guide RNA
PAM: Protospacer-adjacent motif
APC: Adenomatous polyposis coli
dCas9: Inactivated or ‘dead’ Cas9
indels: Small insertions and deletions
KRAS: Kirsten rat sarcoma viral oncogene homolog
β-catenin: Beta-catenin
Wnt: Wingless/Integrated
miRNA: MicroRNA
CDK7: Cyclin-dependent kinase 7
MCF7: Michigan Cancer Foundation –7
ZFNs: Zinc finger nucleases
TALENs: transcription activator-like effector nucleases
BRCA1: Breast cancer susceptibility gene
TET1: Ten-Eleven Translocation-1
5-mC: 5-methylcytosine
5-hmC: 5-hydroxymethylcytosine
IL1R1: Interleukin receptors
TNFR1: Tumor necrosis factor α receptor
HPV: Human papillomavirus
UTR: Un-translated region
NSCLC: Non-small cell lung cancer
HER2: Human epidermal growth factor receptor 2
MAPK: Mitogen-activated protein kinase (MAPK)
ERK: Extracellular signal-regulated kinase
F1H1: Distal 59-flanking region F1, haplotype 1
PARP: Poly(ADP-ribose) polymerase
DYRK2: Dual-specificity tyrosine-regulated kinase 2
ER: Estrogen Receptor
AI: Aromatase inhibitors
MIEN1: Migration and invasion enhancer
PTEN: Phosphatase and tensin homolog
ILC: Invasive lobular breast carcinoma
SIRP-α: Signal-regulatory protein alpha
CD47-SIRPα: Cluster differentiation 47- signal-regulatory protein alpha
TCR: T-cell receptor
CAR T cell: Chimeric antigen receptor T cell
KRAB: Krüppel-associated box
H3K9me3: Histone3 lysin 9 triple methylation
SRC-1: Steroid receptor coactivator 1
qPCR: Quantitative PCR
TP53: Tumor protein 53
PPABLG: α-helical polypeptide
P-HNPs: A PEGylated nanoparticles based on the cationic α-helical polypeptide poly(γ-4-((2-(piperidin-1-yl)ethyl)aminomethyl)benzyl-l-glutamate
DSB: Double strand break
FokI: Endonuclease isolated from *Flavobacteria okeanokoites* I
MYBPC3: Myosin Binding Protein C, Cardiac
E7: Early viral protein
